# Strength Predictive Modelling of Soils Treated with Calcium-Based Additives Blended with Eco-Friendly Pozzolans—A Machine Learning Approach

**DOI:** 10.3390/ma15134575

**Published:** 2022-06-29

**Authors:** Eyo U. Eyo, Samuel J. Abbey, Colin A. Booth

**Affiliations:** 1 Faculty of Environment and Technology, Department of Engineering, Design and Mathematics, Civil Engineering Cluster, University of the West of England, Bristol BS16 1QY, UK; samuel.abbey@uwe.ac.uk; 2 Faculty of Environment and Technology, Centre for Architecture and Built Environment Research, University of the West of England, Bristol BS16 1QY, UK; colin.booth@uwe.ac.uk

**Keywords:** machine learning, artificial intelligence, pozzolans, cement, gradient boosting, soil stabilisation, rice husk ash, palm oil fuel ash, unconfined compressive strength

## Abstract

The unconfined compressive strength (UCS) of a stabilised soil is a major mechanical parameter in understanding and developing geomechanical models, and it can be estimated directly by either lab testing of retrieved core samples or remoulded samples. However, due to the effort, high cost and time associated with these methods, there is a need to develop a new technique for predicting UCS values in real time. An artificial intelligence paradigm of machine learning (ML) using the gradient boosting (GB) technique is applied in this study to model the unconfined compressive strength of soils stabilised by cementitious additive-enriched agro-based pozzolans. Both ML regression and multinomial classification of the UCS of the stabilised mix are investigated. Rigorous sensitivity-driven diagnostic testing is also performed to validate and provide an understanding of the intricacies of the decisions made by the algorithm. Results indicate that the well-tuned and optimised GB algorithm has a very high capacity to distinguish between positive and negative UCS categories (‘firm’, ‘very stiff’ and ‘hard’). An overall accuracy of 0.920, weighted recall rates and precision scores of 0.920 and 0.938, respectively, were produced by the GB model. Multiclass prediction in this regard shows that only 12.5% of misclassified instances was achieved. When applied to a regression problem, a coefficient of determination of approximately 0.900 and a mean error of about 0.335 were obtained, thus lending further credence to the high performance of the GB algorithm used. Finally, among the eight input features utilised as independent variables, the additives seemed to exhibit the strongest influence on the ML predictive modelling.

## 1. Introduction

Ground improvement using the technique of shallow or deep soil mixing has received much interest and acceptance in recent years largely due to its extensive applications in construction projects. In the UK, EU and US where the uptake and implementation of the technology has increased exponentially over the past three decades, environmental policies and laws, taxes, landfill directives and the ever-increasing cost of excavating and moving poor soils has made this method of ground stabilisation even more imminent [1,2,3,4].

Cementitious materials, such as cement and lime, have been used traditionally over the past 50 decades as hydraulic binders to stabilise poor soils. However, the attendant negative environmental impacts associated with the production of these energy intensive binders are a present global concern. Hence, based on current developments in knowledge and research, attention is gradually shifting from an over-reliance on solely cement and lime to the utilisation of waste materials, industrial and agricultural by-products, organics, etc., in soil stabilisation [5,6].

Agro-based environmentally friendly pozzolanic materials, such as rice husk ash, palm oil fuel ash, bagasse ash, coconut shell ash, coconut husk ash, corn cob ash, almond shell ash, etc., have gained considerable attention in soil stabilisation given the ever-growing costs of their disposal [7,8,9,10,11,12,13,14,15,16,17,18]. The major chemical composition of these plant-based pozzolans are alumino-silicates [19,20,21]. Moreover, in order to achieve the desired effect on the mechanical properties of soils, most applications in soil stabilisation have tended towards the partial substitution of calcium-based agents by agro-based pozzolans. The soil-binder mix in this regard can speed up the rate of development of the calcium alumino-silicate hydrated gel (CAH or CASH) as well as the sodium alumino-silicate hydrated gel (NASH) [22]. These binding gels will develop inside the soil voids, and aid in the formation of a more compact soil-binder mix and thus enable a further improvement in the strength of the stabilised soil. Approximately 50–80% reduction in the quantity of calcium-oxide-based additives as a result of the addition of agro-based pozzolanic materials has been reported [23]. Important literature surveys reflecting the composite mix of calcium-based and agro-based agents used in soil stabilisation were carried out recently [15,24,25]. Table 1 is an indication of some of the binders used in the recent past and the target strength properties considered in the improvement.

In general, the determination of the strength properties of soils stabilised by using a composite binder mixture is often a crucial first step towards establishing the correct design mix guideline for field application [26,27,28]. For soils stabilised by using multiple binder combinations, the challenges of establishing a parameter, such as the UCS, may require laborious laboratory experimentation and time-consuming trial batching of binder type, quantities, optimal combinations, choice of curing duration, and the determination of other influencing factors. 

Moreover, conventional techniques of predicting or modelling the UCS of stabilised soils do rely essentially on relationships that are developed empirically from statistical concepts, employing mostly linear, and occasionally nonlinear regression methods [29,30,31]. The equations generated analytically from these methods do tend to determine several unknown coefficients that may affect relationships between the dependent and independent features or variables. Hence, the resulting models, although effective in certain situations, are inherently riddled with shortcomings due mainly to the complexities of the stabilised soil mix. 

In recent times, artificial intelligence paradigms relying on several machine learning (ML) techniques have begun to gain traction as alternatives for the determination of the UCS of soils [32,33,34]. That notwithstanding, the adoption of ML methods for the predictive modelling of improved ground properties has only been reported in a few studies [32,35,36,37,38,39,40,41,42]. Moreover, an application that involves predictive modelling of compressive strength of soils stabilised by eco-friendly pozzolans enriched by cementitious additives has not been reported.

In this study, the gradient boosting (GB) machine learning technique is utilised for the predictive modelling of compressive strength of soils stabilised by cementitious additive-enriched eco-friendly pozzolans. This research shall take into account both regression and multinomial classification of the compressive strength of the stabilised soils. Rigorous sensitivity-driven diagnostic testing to validate the algorithm used and the corresponding statistical outcomes are also undertaken. Finally, it is recommended that an implementation of the concepts derived from this study be applied during the preliminary stages of soil stabilisation for civil construction and related ground improvement applications.

**Table 1:** Studies on soil stabilisation using agro-based and cementitious additive blends.

Pozzolanic Ash	Cementitious Additive	Target Strength	Reference
Rice husk ash	Lime	CBR	[43]
Bagasse ash	Lime	UCS, CBR	[44]
Rice husk ash	Cement, Lime	UCS	[45]
Rice husk ash	Cement, Lime	UCS, Shear	[23]
Bagasse ash	Lime	CBR	[46]
Rice husk ash	Cement	UCS, CBR	[47]
Palm oil fuel ash	Lime	UCS	[21]
Rice husk ash	Lime	CBR, shear	[48]
Bagasse ash	Calcium carbide	UCS, shear	[49]
Rice husk ash	Cement	UCS, tensile strength, flexural strength	[50]
Rice husk ash	Cement	CBR	[51]
Sawdust ash	Lime	CBR	[17]
Coconut shell ash, coconut husk ash	Lime	UCS, CBR	[52]
Palm oil fuel ash	High calcium pulverized fuel ash	UCS, CBR	[53]
Rice husk ash	Cement	UCS, tensile strength	[54]
Palm oil fuel ash, rice husk ash	Calcium carbide	UCS, shear	[55]
Rice husk ash	Lime	UCS, shear, tensile strength, CBR	[56]
Rice husk ash	Lime	UCS, CBR	[7]
Rice husk ash	Lime	UCS, CBR	[57]
Bagasse ash	Cement	UCS, CBR	[58]
Rice husk ash	Lime	UCS, shear, CBR	[59]
Palm oil fuel ash	Cement	Shear	[60]
Rice husk ash	Cement	Shear, CBR	[11]
Rice husk ash	Lime	UCS	[61]
Rice husk ash	Lime	CBR	[62]
Bagasse ash	Lime	UCS, CBR	[63]
Rice husk ash	Lime, calcium chloride	UCS, CBR	[64]
Corn cob ash	Calcium carbide	UCS	[65]
Bagasse ash	Cement	UCS	[66]
Almond shell ash	Lime	UCS	[67]
Plant ash	Cement, calcium chloride	UCS	[14]
Rice husk ash	Cement, lime	UCS	[35]

## 2. Methodology

### 2.1. Database Development, Pre-Processing, and Exploratory Analysis

**Figure 1:**
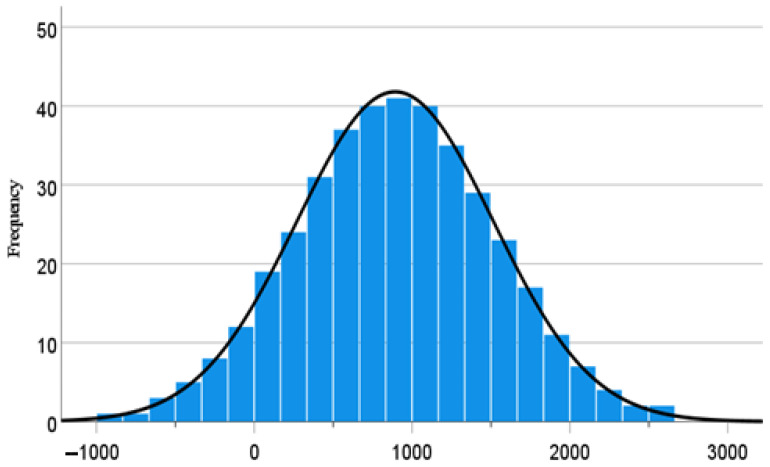
Normal distribution of values of UCS.

A dataset of 392 soils stabilised using cementitious additives’-enriched agro-based pozzolans in various proportions and combinations, compacted and cured for 7, 14 and 28 days were compiled from a very intensive literature search [7,17,21,22,35,44,47,49,50,53,54,55,56,63,67]. As stated previously, most agro-based pozzolanic materials are composed mainly of alumino-silicates. The range of some of the main chemical compositions of the pozzolans and those of the cementitious materials (containing mostly of calcium-oxide compounds) utilised to stabilise the soils are given in Table 2. A very broad range of agro-based pozzolans (rice husk ash, palm oil fuel ash, bagasse ash, coconut shell ash, coconut husk ash, corn cob ash, and almond shell ash) were used to stabilise the soils. On the other hand, the cementitious materials consist of cement, lime, cement kiln dust, high calcium fly ash and calcium carbide. As could be observed in Table 2, X-ray fluorescence (XRF) measurements conducted on the binding agents used in this study indicates the maximum proportion of alumino-silicates in the agro-based pozzolans as being about 93%, while that of cementitious additive is about 25%. On the other hand, the highest amount of calcium oxide compound realized in the pozzolanic ashes is about 14% compared to 95% in the cementitious binders. It seems from Table 2 that the proportions of both agents that can be used to stabilize the soil could be an important trade-off between their innate chemical compositions, the target compressive strength to be achieved and the impact of their usage on the environment. It is also pertinent to bear in mind that the clay soils to be stabilized are themselves mostly siliceous as indicated by their chemical compositions in Table 2; hence, they should be taken into account in deriving a suitable design mix. Standard preparation methods including those involving slight modifications of traditional or standardized measurement procedures carried out to reflect special laboratory testing conditions were followed to achieve the aims of stabilisation. Since the nature of the dataset of UCS are diverse in this regard, it was necessary to normalise these data in order to enhance the significance of the overall modelling and the reliability of the results of findings. A two-step inverse-normal data transformation approach was applied on the dataset of UCS regarded in this study as the target variable [68]. As could be observed from Figure 1 and Table 3, normally distributed data and relatively lower values of kurtosis (−0.16) and skewness (1.63 × 10^−6^) suggest that the dataset can be reliable for ML modelling.

**Figure 2:**
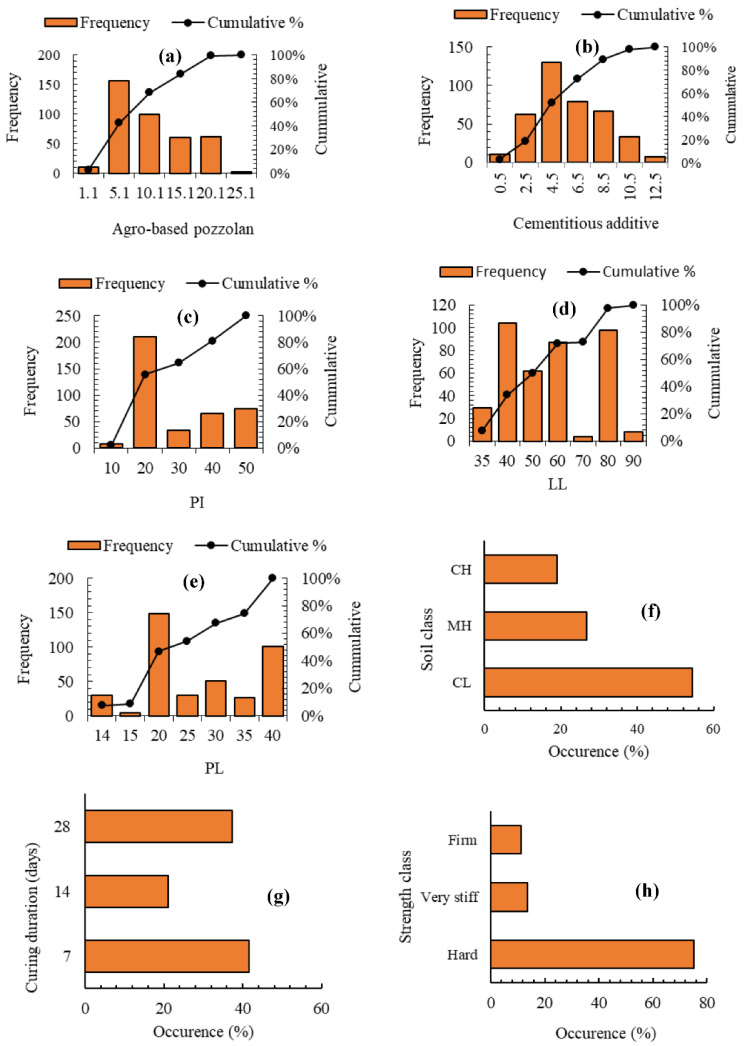
Distribution of input features for (**a**) Agro-based pozzolans; (**b**) Cementitious additives; (**c**) Plasticity index (PI); (**d**) Liquid limit (LL); (**e**) Plastic limit; (**f**) Soil class; (**g**) Curing duration; (**h**) Strength class.

A total of 8 independent variables are used as input features in the ML modelling namely, values of agro-based pozzolans, cementitious additives, soil class, liquid limit, plasticity index, plastic limit, curing duration and strength class. Exploratory dataset analysis carried out on these variables yields the statistical metrics and distributions shown in Table 3 and Figure 2, respectively.

Given that the independent variable dataset will be used in its raw form for the ML modelling, it is very refreshing to note how reasonably low their kurtosis and skewness scores are as observed in Table 3. It is very necessary to use raw independent data of the variables in the predictive modelling to preserve as well as ensure an accurate representation of the natural reality of random distributions. As Table 3 shows, the range and proportion (calculated by weight of dry soil) of the agro-based pozzolans (lowest of 0.1% and highest of 25%) and the cementitious materials (lowest of 0.5% and highest of 11.25%) used, demonstrate a very diverse mix of binder quantities that are used to stabilise the soils. Except for the soil plasticity properties, Figure 2a,b shows a fairly uniform frequency distribution for the binders used. The distribution of the soil class is observed as being skewed to favour mostly soils of lower plasticity. However, there is almost a satisfying balance between the soil classes if both high plasticity classes (CH and MH) are considered together compared to the lower plasticity class (CL). The frequency distribution of curing duration (Figure 2g) seems fair, except for the UCS strength classes, which, according to Figure 2h, is imbalanced and skewed towards the hardened stabilised soils. Thus, the frequency distribution for the stabilised soil’s compressive strength class (or consistency) in this regard indicates that most of the stabilised soils in the dataset were greater than approximately 400 kPa, as Table 4 shows. The UCS class shall serve as a target (or dependent) variable in the multinomial classification ML prediction, while, as already stated above, the actual numerical values of the UCS shall be used as dependent variables in the ML regression modelling considered subsequently in this research.

### 2.2. Gradient Boosting Machine (GBM)

Boosting is generally an ensemble machine learning technique that involves an aggregation of based learners to enable better predictions of mostly classification and regression problems. Gradient boosting (GB) machine works by optimising a differentiable loss function (an example is the ‘squared error’ for regression and ‘logarithmic’ for classification) as well as an additive modelling that involves taking a weighted sum of several suitable base learners in order to minimise the loss function. In its simplest from, the GBM as an additive model can be represented mathematically as

$$F_{m}\left(X\right)=F_{m-1}\left(X\right)+\eta \cdot f_{m}\left(X\right)$$

where *F* = ensemble model, *f* = base(weak) learner, *η* = rate of learning or shrinkage and *X* is the input vector.

*F_m_* (*X*) is the result of each iteration obtained by minimising a loss function and therefore can be considered as a directional vector (*r_m−_*_1_), which points to the steepest decent. Hence, the GB machine can then be expressed alternatively as

$$F_{m}\left(X\right)=F_{m-1}\left(X\right)+\eta \cdot r_{m-1}$$

If the function that is being approximated is given as

$$F\left(x\right)=f_{1}\left(x\right)+f_{2}\left(x\right)+f_{3}\left(x\right)+\cdots +f_{n}$$

If there are *n* number of samples in a dataset (*D*), with each sample having *m* set of features in a vector *x* and a real target or dependent value of *y* all expressed as

$$D=x_{i},y_{i}\left(\left|D\right|=n,x_{i}∊\;{\mathbb{R}}^{m},y_{i}\;∊\;\mathbb{R}\right)$$

Then, an ensemble of trees considering additive modelling can be given as below:

$$y_{i}=\phi \left(x_{i}\right)=\sum_{m=1}^{M}f_{m}\left(x_{i}\right),f_{m}\;∊\;ℱ$$

where *M* = number of base leaners and ℱ = regression tree space.

Additionally, if the differentiable loss function is given as

$$ℒ\left(\phi \right)=\sum_{i}l(\hat{y_{\iota }},\;y_{i)}$$

then the first step in initialising the model with a constant value by minimizing the loss function becomes

$$F_{0}\left(x\right)=\underset{\rho }{\underset{︸}{\arg\min}}\sum_{i=1}^{n}L(y_{i},b_{0})$$

where *b*_0_ = minimisation of loss function at 0th iteration.

For *m* = 1 to *M*, the following is computed for all the *n* samples for *i* = 1, …, *n*

$$r_{im}=-\left[\frac{\partial L\left(y_{i},F_{m-1}\left(x_{i}\right)\right)}{\partial F_{m-1}\left(x_{i}\right)}\right]$$

Next, we fit a regression (or classification tree) to *r_im_*, allowing each tree to be denoted by *R_jm_* for *j* = 1, …, *J_m_*, where *J_m_* is the number of leaves in the trees created in the *m^th^* iteration.

For *j* = 1, …, *J_m_* the following is then computed:

$$\rho_{m}=\underset{\rho }{\underset{︸}{\arg\min}}\sum_{i=1}^{n}L(y_{i},F_{m-1}\left(x\right)+\rho_{m}\sum_{j=1}^{J}b_{jm}$$

where *b_jm_* = least square coefficient or the basis function, *ρ_m_* = leaf weight or scaling factor

The equation then simplifies to

$$\rho_{jm}=\underset{\rho }{\underset{︸}{\arg\min}}\sum_{i=1}^{n}L(y_{i},F_{m-1}\left(x\right)+\rho_{m}\cdot b_{m}),$$

With the update given as

$$F_{m}\left(x\right)=F_{m-1}\left(x\right)+\eta \cdot \rho_{m}\cdot \sum_{j=1}^{J_{m}}b_{jm}$$

It is important to note that the parameters or hyperparameters required (among other factors) will have to be carefully selected in order to obtain an optimised or desirable results of the ML prediction. The following section shall describe the methods adopted to optimise the GB machine to ensure higher performance on multinomial classification and regression.

### 2.3. Model Optimisation 

#### 2.3.1. Hyperparameter Tuning 

In order to ensure the best performance of the GB model, a series of stepwise-randomized searching were implemented to select the best performing hyperparameters using python’s sklearn searching class type called ‘‘RandomizedSearchCV”. Table 5 shows the hyperparameters eventually chosen to optimise the algorithm on both the training and testing datasets.

#### 2.3.2. Cross-Validation

The *k*-fold cross-validation technique was applied to enhance learning and validation on 80% of the dataset. Cross validation also ensures that undue overfitting of the algorithm on the training set was prevented. After several trials, 10-fold cross validation was regarded as the most effective in the ML modelling. It is important to note that a further 20% of the data was set aside for model testing.

### 2.4. ML Performance Evaluation Metrics

For an assessment of the performance of the ML model on the multinomial classification problem, accuracy, precision, recall and F1 score were used. Additionally, in order to depict the capacity of the model to predict the probability of the compressive strength of the stabilised soils belonging to different categories across a specified decision threshold, the receiver operating characteristic curve (ROC) and corresponding area under curve (AUC) were used. ROC is a plot of true positive rate (TPR) or sensitivity versus false positive rate (FPR) (or one less specificity) under some threshold values hence, separating “noise” from “signals”. AUC is a measure of the actual ability of a model to distinguish between class labels. For the regression problem, coefficient of determination (R^2^) and mean absolute percentage error (MAPE) metrics shall be adopted.

## 3. Results and Discussion

### 3.1. Behaviour of the Stabilised Soils

**Figure 3:**
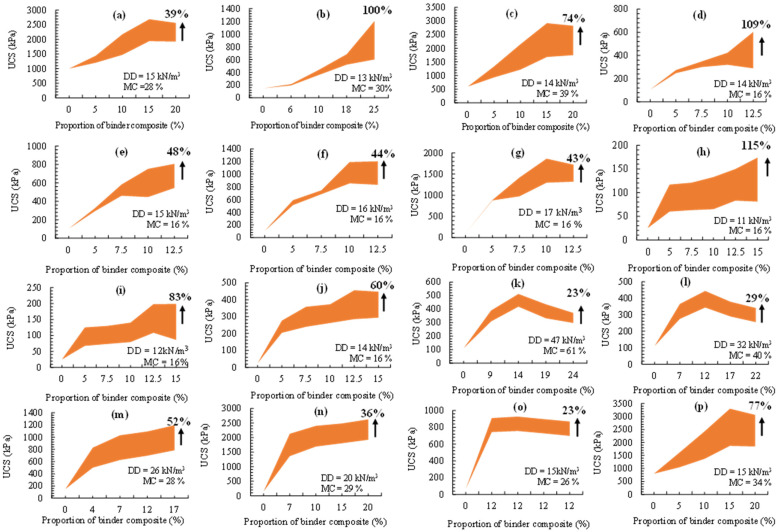
Variation of UCS of stabilised soils with duration and binder proportion. (**a**) 39% increase in UCS; (**b**) 100% increase in UCS; (**c**) 74% increase in UCS; (**d**) 109% increase in UCS; (**e**) 48% increase in UCS; (**f**) 44% increase in UCS; (**g**) 43% increase in UCS; (**h**) 115% increase in UCS; (**i**) 83% increase in UCS; (**j**) 60% increase in UCS; (**k**) 23% increase in UCS; (**l**) 29% increase in UCS; (**m**) 52% increase in UCS; (**n**) 36% increase in UCS; (**o**) 23% increase in UCS; (**p**) 77% increase in UCS.

The UCS of soils stabilised using cementitious additives-enriched pozzolanic materials and compacted at optimal conditions (dry density (DD) and moisture content (MC)) as shown in Figure 3. The stabilised materials were thoroughly synthesized and slightly modified from their original sources with the horizontal axis showing a combined total of the binders (agro-based pozzolans and cementitious additives) used (by weight of the dry soil). Generally, a rising trend is observed as represented by the shaded bands signifying increments up to 28 days of curing. The increase in strength corresponds to an increase in the proportion of the binders with the curing duration. However, a few slight deviations from this trend are also observed, where there seems to be a threshold signifying the highest rise in UCS corresponding to the optimal binder mixes beyond which there is a minimal decrease in UCS as the total binder proportion increases (Figure 3a,c,g,k,l,p). 28-day strength development of twice and slightly more than doubled those observed at 7 days of curing are noticed in Figure 3b,d,h. As indicated in Figure 3b, 100% strength increase at 28 days, up from 7 days of curing, is also realised when the binders with combined proportion of 25% are used to stabilise the soil. On the other hand, the strength increases of about 23% (from 7 to 28 days of curing), considered the least of all, are indicated in Figure 3k,o.

In general, the total amount of agro-based pozzolans combined with calcium-based additives required to stabilise the soil to produce the highest strength is approximately 15% by weight of the dry soil. Moreover, it has been reported that the proportion of agro-based pozzolanic ashes should be more than calcium oxide-rich additives in the stabilised soil in order to achieve the desired aim of reducing carbon footprinting [51,54,69]. It should also be noted that although the behaviour of the stabilised soil in terms of strength increase is mostly due to the proportion of binders used and curing duration, the methods of preparation of the stabilised soil, curing conditions, method of compaction, soil type, laboratory instrumentation, etc., can also contribute to this trend. It is suggested that these secondary factors be further investigated and modelled to include environmental factors (temperature, freezing and thawing, wetting and drying, etc.), which could also have potential effect in terms of the durability of a stabilised soil.

### 3.2. ML Classification of UCS

Three compressive strength categories or classes (‘firm’, ‘very stiff’ and ‘hard’) are obvious from the dataset as earlier indicated by their statistical distribution in Figure 2h. Hence, an application of ML multiclass classification was used to gauge the performance of the GB ML algorithm by investigating its capacity to properly learn the stabilised soil strength patterns and therefore provide predictions accordingly. In most cases, it is very essential that for multinomial classification problems, a threshold point be set for the classifier’s boundary across the different categories. Hence, a sensitivity analysis was performed by using the ROC (receiver operating characteristics curve) and resulting area under curve (AUC) as relevant tools for the assessment of the capacity of a ML algorithm to identify or decern the true positives among the class labels.

**Figure 4:**
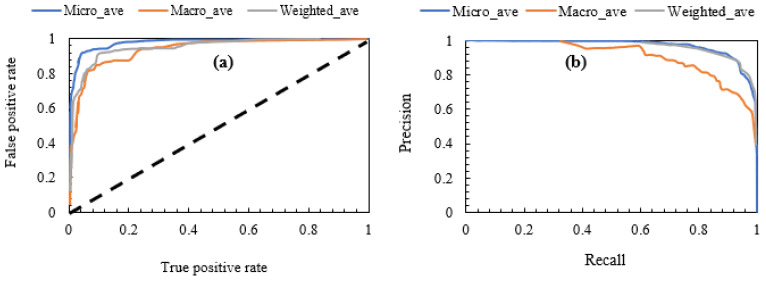
Classification metrics. (**a**) ROC curve, (**b**) precision–recall curve.

As shown in Figure 4, the GB algorithm possesses a very high capacity to identify the positive and negative UCS consistency classes. Table 6 also shows very high values of the multinomial classification metrics resulting from the ML prediction. With a ‘non weighted’ accuracy of 0.920, it can then be inferred that the ability of the GB ML algorithm in the multiclass prediction to ensure that both type 1 and type 2 prediction errors have been kept at the barest minimum has been achieved. Table 6 also shows that the assignment of weighted average to take care of under-represented class instances does clearly improve the individual multiclass performance with ‘weighted’ accuracy of 0.966 compared to the overall accuracy metric of 0.920. Further, it is important to note that the averaging that considers each class instances (i.e., micro-averaging) gives higher scores when compared to the averaging, whereby all equal class instances (i.e., macro-averaging) are taken into account with respect to the most frequently occurring class labels. Additionally, by comparing other metrics, such as the recall rates and precision scores, it is observed in Table 6 that using micro-averaging in this regard does provide relatively higher sensitivity and thus better performance.

**Figure 5:**
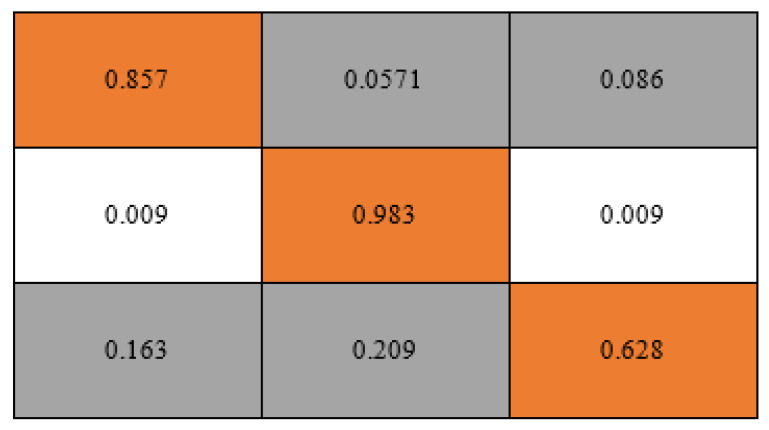
Normalised confusion matrix of multinomial classification.

The number of misclassified instances indicated in the normalised confusion matrix could have been due to an imbalance of the strength categories or classes. However, it is very important to note that in order to ensure an overfitting of the data by the algorithm was prevented, a 10-fold cross validation was used. The distribution of the strength categories as earlier indicated in Figure 5 indicated that most of the stabilised soils in the dataset were classed as being ‘hard’ (i.e., >400 kPa). Hence the prediction has unsurprisingly shown slight biases towards the stabilised soils’ ‘hard’, ‘very stiff’ and ‘firm’ categories in that order. Based on the nature of dataset used in this study, this behaviour of the ML model is also partly a reaffirmation of the effectiveness of the eco-friendly pozzolanas combined with the cementitious binders in stabilising the soft soils. A total of 12.5% misclassified instances was achieved.

Another means of validating a classifier’s performance is by using the cumulative gains (CG) and its corresponding lift curve (LC). By relying on the CG or LC, further justification can be provided to show the classifier’s effectiveness in the ML prediction especially when compared to another model exhibiting an act of random guessing between multinomial categories. LC is extrapolated from the CG and represents the cumulative gains between a model that also includes the baseline lifting portion (or horizontal percentile axis) of the curve. The lift is viewed mathematically as the ratio of 1 s on a certain sampled data point, to that of 1 s on the entire dataset as given in the equation below. This then can be seen as the predictions that a random model would be making.

$$\mathrm{lift}=\frac{\mathrm{prediction}\;\mathrm{rate}}{\mathrm{average}\;\mathrm{rate}}=\frac{\mathrm{ratio}\;\mathrm{of}\;1\;s\;\mathrm{in}\;a\;\mathrm{particular}\;\mathrm{samples}\;\mathrm{dataset}}{\mathrm{ratio}\;\mathrm{of}\;1\;s\;\mathrm{in}\;\mathrm{the}\;\mathrm{whole}\;\mathrm{dataset}}$$

**Figure 6:**
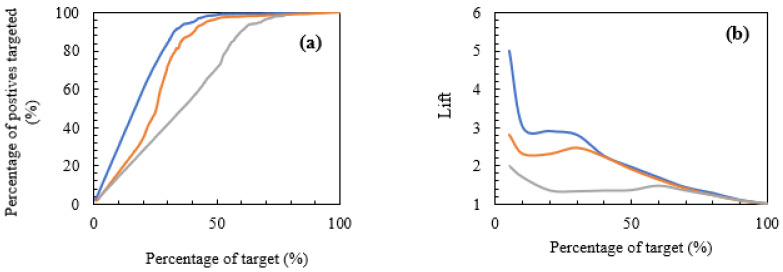
Multinomial classification graphs. (**a**) Cumulative gains curve, (**b**) lift curve.

Figure 6 shows the CG and LC of the multiclass classification. A high lift indicates a high performing algorithm. The lift indicated by micro-averaging indicates the highest rising from the baseline percentile axis up to the value of about 5 as seen in Figure 6. Although LC could also be used to compare two or more models, the performance of GB in this regard can be viewed as very reasonable. Hence, if 5% of the sampled data is considered, it therefore means that there are 5 times more positive class labels for each of the classes than an average. Put differently, for 5% of the dataset sampled, about 50% of the entire dataset would contain all the positive class labels.

It is pertinent to note that the performance of the ML model as indicated by the LC resulting from micro-averaging is generally higher than those of macro- and weighted lift scores. This behaviour confirms further, the multiclass ML prediction outcomes given by the ROC and CG curves.

### 3.3. ML Regression of UCS

**Figure 7:**
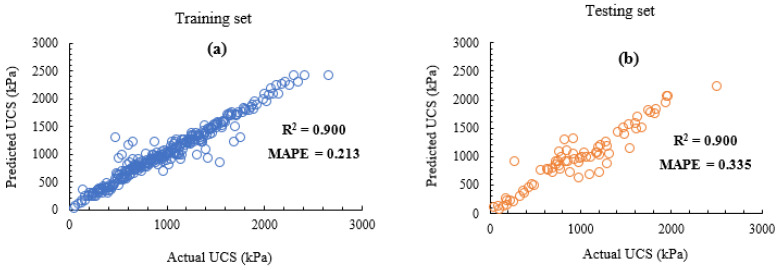
Regression curves. (**a**) Training set, (**b**) testing set.

GB demonstrates a very high capacity to learn the complexities of the soil stabilised using the different additives and their various combinations as already seen from the multiclass classification above. As can be observed from Figure 7, both the coefficient of determination for the train and test sets are equal at 0.900. Further evidence of this high performance is indicated by the very low percentage error of both the training and testing datasets being 0.213 and 0.335, respectively. Tabarsa et al. [35], in their studies utilising both artificial neural networks and support vector machine algorithms, obtained a coefficient of determination of about 0.990, albeit on a smaller number of dataset (137 datapoints). Unlike the present study, they did not utilise the technique of cross validation, and hence, overfitting of the dataset could not be ascertained. Additionally, Tabarsa et al. [35] only considered a single agro-based ash (rice husk ash) and two cementitious binders (cement and lime) in the stabilised soil mix, whereas the present study utilised a multitude of the agro-based and calcium-oxide based agents in the soil stabilisation. While this could mean that the stabilised soils’ model in this study is more non-linear, it is also important to add that the eight input variables utilised in the model may have also impacted the non-linearity of the model compared to six such input features used in the research conducted by Tabarsa et al. [35].

It is important to emphasise that cross-validation was performed on the training dataset used in this study in order to prevent undue overfitting of the model. Further sensitivity analyses to justify the capability of the GB algorithm used are discussed in the sections following.

### 3.4. ML Model Diagnostics

#### 3.4.1. GB Model Residuals

**Figure 8:**
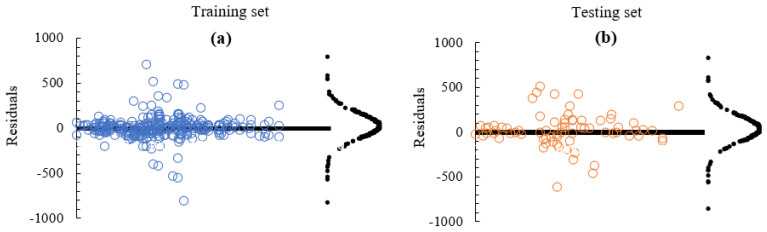
Residual curves. (**a**) Training set, (**b**) testing set.

Residual plots of ML predictions can provide an insight into the behaviour of a model while also offering the means of validating such a model. By utilising the residual plots, a ML model’s predicted or observed errors can be assessed and evaluated in order to judge its consistency with that of its corresponding stochastic errors (i.e., its measure of unpredictability or randomness). Figure 8 indicates the level of independence of the residuals for both the training and the testing sets when applying GB ML model. As could be observed, ML predictions on the dataset have demonstrated very high independence of the residuals or stochastic prediction errors, given that the training and testing dataset are relatively very close and concentrated around the zero-line including a very random distribution of the data points. Almost similar trends of very high symmetry about the origin are exhibited by both training and testing sets of data thus further confirming their matching R2 metric score as indicated above followed by a minute difference in the mean absolute percentage error metric. That notwithstanding, as could be observed from the residual plot of Figure 8, a few data points seem to be far away from the zero-line, which also confirms that the residuals may not be entirely independent.

#### 3.4.2. Distribution of Residuals

**Figure 9:**
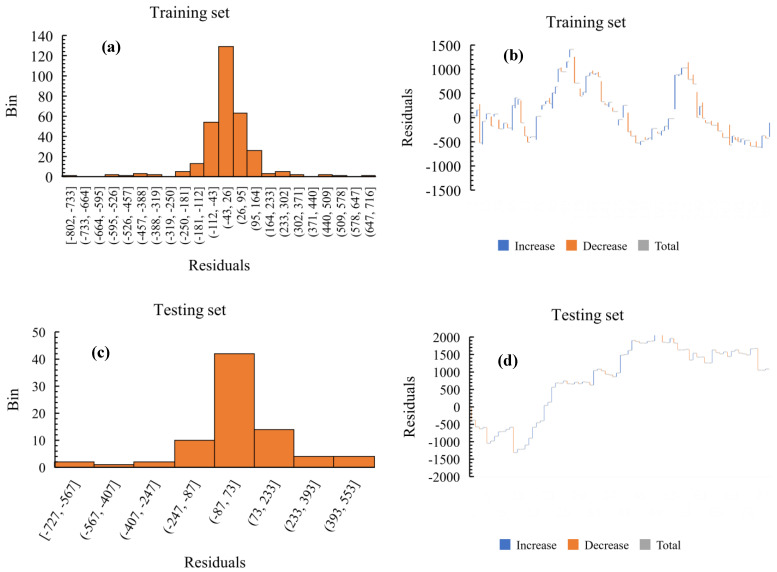
Distribution of residuals. (**a**) Normal distribution for training set, (**b**) cumulative curve of training set, (**c**) normal distribution for testing set, (**d**) cumulative curve of testing set.

The normal frequency distribution plot of the residuals is another means for which a ML algorithm’s authenticity and effectiveness can be assessed. Figure 9 shows the cumulative distributions on histogram plots of the GB regression on training and testing sets. Much like the residual plots given above, there is a higher symmetry with very balanced distributions of the GB model predictions about the centre, although the residuals of the training sets are slightly higher, given they contain most of the data points compared to the testing set. Furthermore, a very high performing model will tend to possess residuals with peaks at the centre or the origin in some cases but with only marginal stochastic errors at the seams, whereas an inaccurate model with very low predictive performance will tend to have much higher errors at the seams and fewer peaks at the centre of the distribution. Based on this concept, it could be observed that although GB prediction on the training set does possess a higher residual peak given the greater quantity of the dataset used, both it and the predictions on the testing set tend to have very low residuals at the extremes of the distribution, hence indicating further the high level of accuracy provided by the model in the ML prediction.

Overall, when the algorithm is applied on the training dataset set, the total of residual data points is about 0.04% of each of the sum of the predicted and actual data (Figure 9b). Meanwhile, ML learning regression using the testing set would produce a total of approximately 1.60% of the sum of the predicted and actual data (Figure 9d). Although the performance of the ML algorithm on both the training and testing set are equal when considering the regression coefficient of 0.900 for both sets of data, further diagnostics has revealed that the errors (although very marginal) inherent in the predictive model are slightly higher on the testing dataset than on the training set. This is also a confirmation of their different mean percentage error scores as observed previously.

### 3.5. ML Model Interpretation

#### 3.5.1. ML Feature Importance

**Figure 10:**
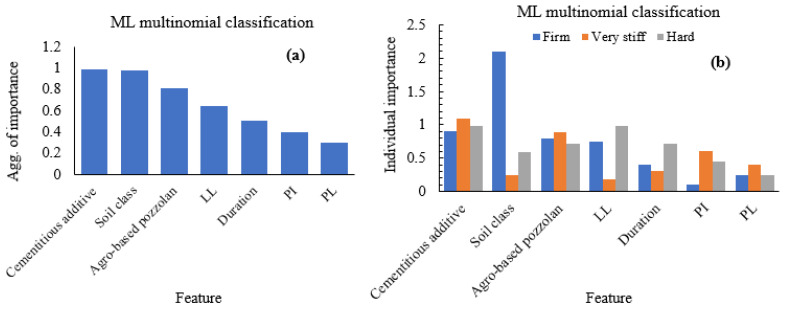
Multinomial classification feature importance. (**a**) Aggregate importance, (**b**) individual importance.

Indicators of the importance of input features or independent variables in ML prediction can provide an insight into the dataset utilised in the stabilised soil modelling, while also enabling in some instances an improvement of the effectiveness of the adopted algorithm in the preliminary stages of the model selection. The relative usefulness and significance of the input features used in the modelling or forecast of target variables are shown in Figure 10 for both the multiclass classification and ML regression.

For the classification problem, the feature importance of the input variables aggregated over the three target categories (strength classes) shows the cementitious additives as having the greatest influence on the prediction of the strength class. Although the importance of the soil class is greater than that of the pozzolanic materials, it could be concluded generally that the binders used in the soil stabilisation do possess the highest effect on the prediction of the strength class within the bounds of dataset used in this study. When considering the performance of the features in the prediction of each of the different class categories, it is observed from Figure 10 that the binders again do possess the highest significance in the ML classification. In the same vein, the binders are also observed as consistently having an important bearing on the class categories as noted by the similar levels of feature importance values for each of pozzolans and calcium-oxide-rich additives across the three class categories. It is interesting to observe that although the soil class has the second highest importance after calcium-oxide-rich additives, it does not seem to consistently contribute to the performance or multiclass prediction of the strength categories. Again, Figure 10 does also indicate how the soil plasticity features are lower in the ranking of importance in the ML classification.

**Figure 11:**
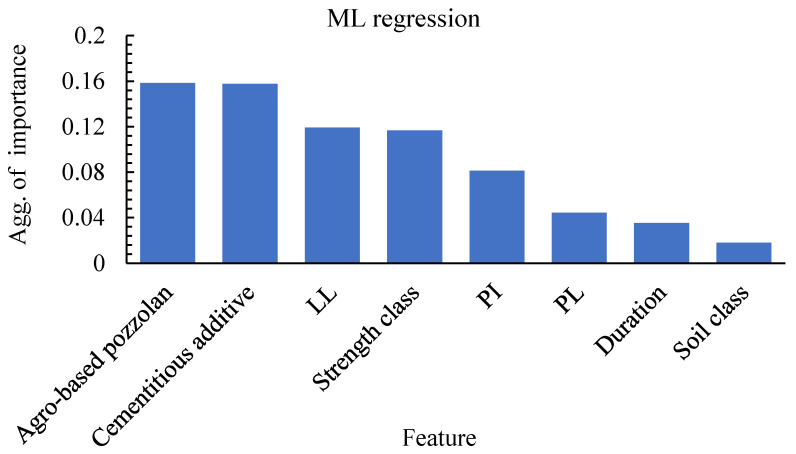
Regression aggregate feature importance.

Almost a similar trend in terms of feature importance in ML classification is demonstrated by the input features when considering ML regression on the actual compressive strength values of the stabilised soils. It is clearly seen from Figure 11 that here, the binders do possess much stronger influence on the prediction. Interestingly, the soil class seems to have the least importance in the prediction of the target variable. It is also very refreshing to note how the soil plasticity properties used as input features in the ML forecast have a stronger bearing on the regression forecast when compared to the ML classification. It is important to stress that although curing duration is an important consideration in determining the strength gain in soil stabilisation, its contribution as an input feature is judged, but the GB model is only fair both in the classification and regression problems. This could be seen as positive at least within the context of the dataset used in this study. This does suggest that the GB ML algorithm can show high performance by determining either what strength class the stabilised soil belongs or what compressive strength it possesses, irrespective of the knowledge of the age of curing.

#### 3.5.2. Partial Dependence and Interaction Plots

A partial dependence plot (PDP) and an individual conditional expectation (ICE) plot could be utilised to analyse and visualise the interaction that exit between the target response variable and a set of explanatory or input variables of interest. PDP indicates the dependence (taken as an average) between the target response variable and a set of independent variables of interest, marginalizing over the average values of the other input variables (or the ‘complementary’ features). Meanwhile, an ICE allows a visualisation of the dependence of a prediction on a variable for each sample when considered separately with one line per sample.

**Figure 12:**
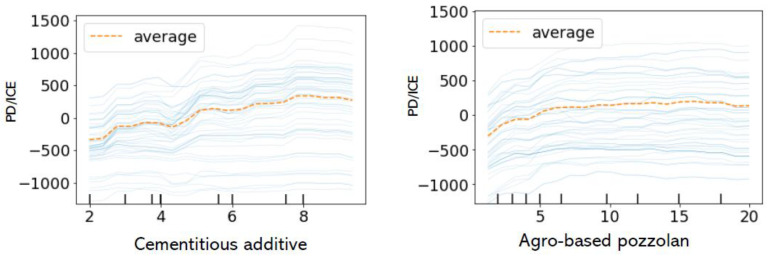

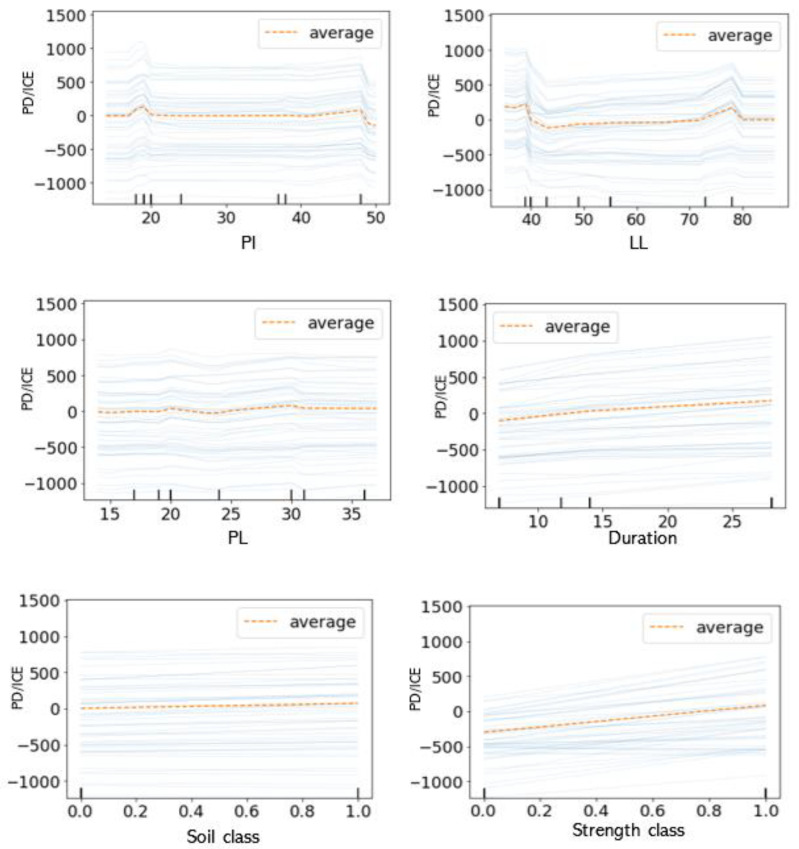
Partial dependence and individual conditional exception plots.

Figure 12 indicates one-way PDP plots with ICE lines between the explanatory features and the response variable. As could be observed, the strongest non-linear relationship seems to exist mostly between the binders (cementitious additive and agro-based pozzolans) and the target or response variable of UCS, although there are some exceptions, where the response variable remains almost constant in some ranges of the average amount of cementitious additive and agro-based pozzolans. This indicates a dependence of the predicted variable on the binders. However, there appears to be almost no relationship between the duration and the strength class with the target variable as indicated by the ICE lines being constant throughout in most ranges of the median of the explanatory features.

**Figure 13:**
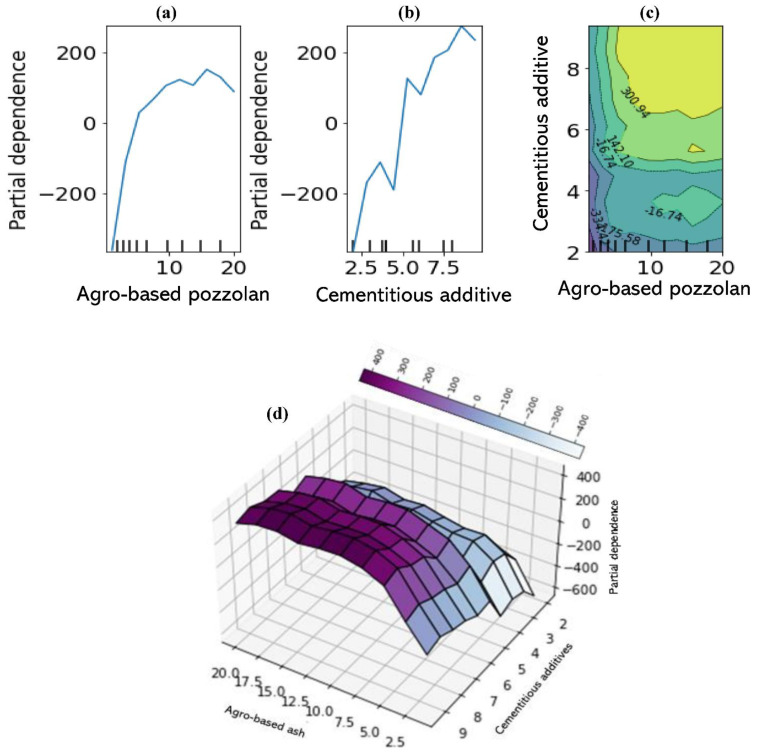
Partial dependence plots: (**a**) one-way dependence for agro-based pozzolan, (**b**) one-way dependence for cementitious additive, (**c**) two-way dependence for additives’ features, (**d**) 3-dimensional two-way dependence for additives’ feature.

**Figure 14:**
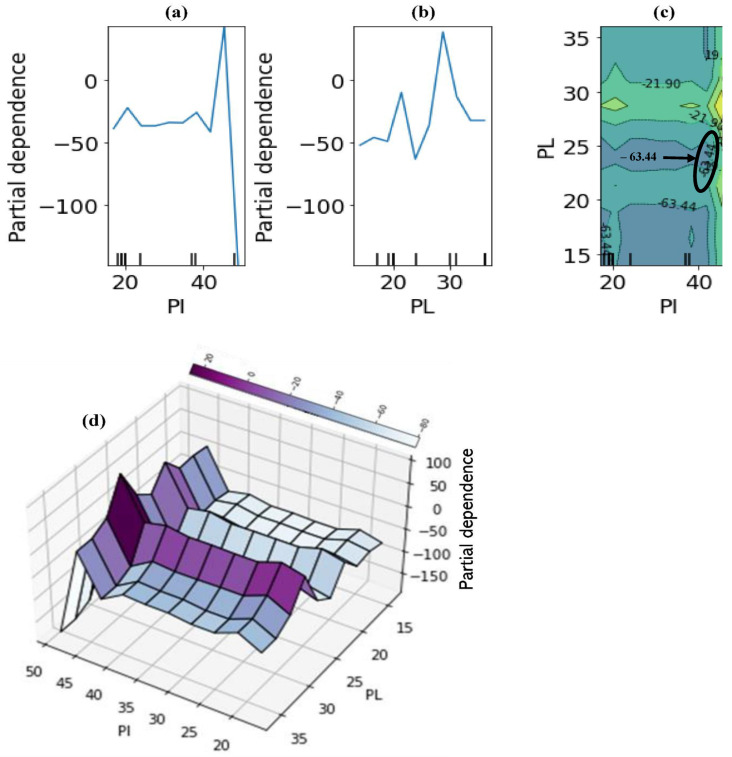
Partial dependence plots: (**a**) one-way dependence for PI, (**b**) one-way dependence for LL, (**c**) two-way dependence for soil plasticity features, (**d**) 3-dimensional two-way dependence for soil plasticity features (Python, version 3.8).

**Figure 15:**
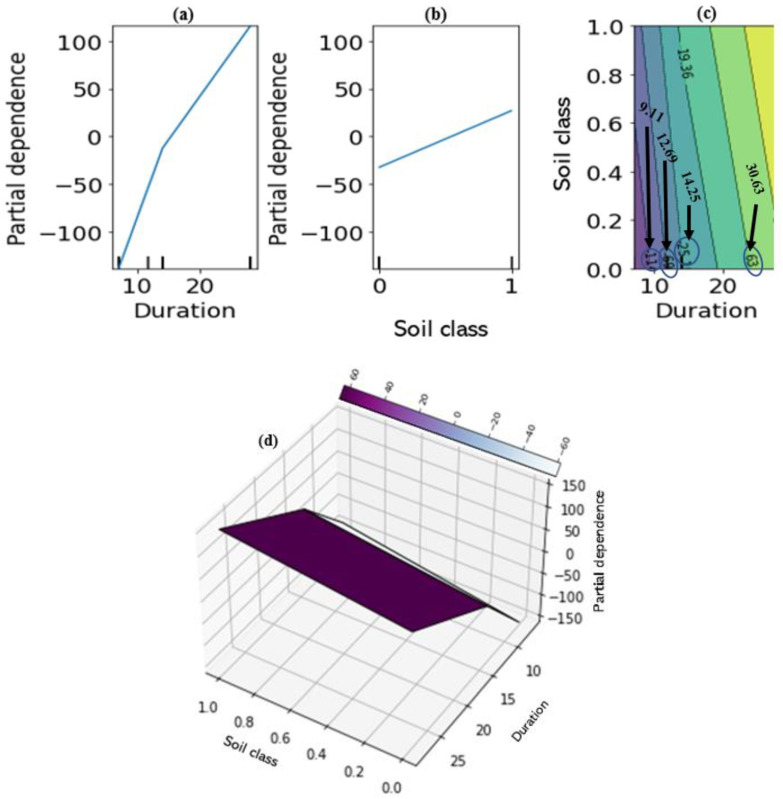
Partial dependence plots: (**a**) one-way dependence for duration of curing, (**b**) one-way dependence for soil class, (**c**) two-way dependence for curing duration and soil class, (**d**) 3-dimensional two-way dependence for curing duration and soil class (Python, version 3.8).

A much better visualisation of the interaction between each of the two most influential and less influential features in the prediction is shown in Figure 13. The two-way interaction PDP plots indicate the dependence of the average of the target variable (UCS) on the joint values of the most important and the less influential variables. When considered together, it can be clearly seen from Figure 13a–d that there is a strong interaction between the two most important features: for an average agro-based pozzolan of between 7 and 15%, the UCS is nearly independent of the cementitious additive used (indicated by the nearly horizontal contours of Figure 13c), whereas for median values between 0 and 7% (indicated by vertical contours), there is a strong dependence on the cementitious additives. Although there are pockets of exceptions to these relationships as observed from Figure 13c, it could be concluded generally that the quantity of agro-based binder required in combination with a calcium-based cementitious agent for high stabilised soil performance in terms of unconfined compressive strength should be limited to about 15% but not less than 7% calculated by weight of the dry soil. With regards to the soil plasticity properties, for an average plasticity index (PI) of less than 40%, the UCS is nearly independent of the plastic limit (PL) (Figure 14a–d). Finally, there seems to be almost no dependence of UCS on the soil class for all average values of the curing duration (Figure 15a–d).

### 3.6. Comparison to Other Tree-Based ML Ensembles

A confirmation of the performance of the GB ML algorithm used in this study when compared to other commonly used tree-based ensemble ML techniques is given in Table 7. GB does clearly outperform its counterparts and base estimators as indicated by the statistical metrics. Granted, these algorithms are characterised by slightly different parameters; however, these were optimised and used for the training and testing. The order of performance and ranking of the algorithms on the stabilised soil dataset is Gradient Boost > Adaboost > Extremely Randomised Trees > Random Forest > Decision Tree.

## 4. Study Significance

The importance of machine learning to civil and environmental engineering design and construction especially in ground improvement works including but not limited to road subgrades, building foundations, embankments and cut slopes, bridge abutments, exclusion barriers, liquefaction mitigation, backfills, contaminated ground remediation, etc. cannot be over- emphasized. The concept of artificial intelligence as applied in this study can save time, cost and money during the planning and design stages of ground improvement. Examples of some of the preliminary exercises that can be circumvented are laborious laboratory experimentation and time-consuming trial batching of binder type, quantities, optimum combinations, choice of curing duration, and the determination of other influencing factors. In order to practically implement the model studied herein, all its resources, including background scripting, would have to be deployed and persisted on an organisation’s server to be used to train and test on new data of stabilised soil’s unconfined compressive strength.

However, it should be borne in mind that only a few of the plethora of factors that can directly or indirectly influence soil stabilisation were considered. Hence, it is suggested that the principles applied in this research be extended to include modelling of not just soil strength behaviours but also other important serviceability design parameters that involve settlement and soil swelling or expansion. Moreover, predictive modelling using artificial intelligence is recommended for stabilised soils subjected to different curing and environmental durability conditions in future studies. It is also suggested that various other environmentally friendly materials be utilised to stabilise the soils and predictions made using machine learning models.

## 5. Conclusions

An artificial intelligent approach using machine learning for predictive modelling of soil stabilised by eco-friendly pozzolans rich in cementitious additives was studied. Sensitivity-driven diagnostic testing was also performed to validate the algorithm used along with the corresponding statistical outcomes. Highlights and the summary of this study are as follows:

- The combined total quantity of agro-based pozzolans and calcium oxide-rich required to stabilise a soil to produce the highest strength is approximately 15% (by weight of the dry soil) but with the pozzolan having the greater amount in the binder mix.
- GB algorithm shows a very high capacity to distinguish between positive and negative unconfined compressive strength categories (‘firm’, ‘very stiff’ and ‘hard’) with an overall accuracy of 0.920, weighted scores of 0.938 and 0.920 for precision and recall rates respectively and an overall lift of 5 in a multinomial classification.
- Although ML multiclass prediction was slightly biases towards the stabilised soils’ ‘hard’, ‘very stiff’ and ‘firm’ categories in that order, only 12.5% of misclassified instances was achieved by the algorithm.
- A coefficient of determination of approximately 0.900 and a mean absolute percentage error of 0.335 lends further evidence to the high performance of the GB algorithm when applied to a regression problem.
- Rigorous diagnostic tests performed on the GB algorithm revealed only marginal inherent stochastic errors in the prediction with the errors only being slightly higher on the testing set when compared to the training set.
- Overall, among the 8 input features used as independent variables, the binders (both agro-based pozzolans and the cementitious additives) tend to exhibit the strongest influence on the ML predictive modelling in the multinomial classification and regression modelling.

## Figures and Tables

**Table 2:** Range of chemical compositions of the materials.

Oxide	Agro-Based Pozzolans	Cementitious Additives	Soils
	(%)	(%)	(%)
Silica (SiO_2_)	9.00–93.15	0.90–25.12	41.45–71.16
Alumina (Al_2_O_3_)	0.21–19.39	0.13–12.20	3.41–19.00
Iron oxide (Fe_2_O_3_)	0.21–6.10	0.15–4.46	3.59–21.34
Calcium oxide (CaO)	0.30–13.87	8.47–95.30	0.15–5.76
Potash (K_2_O)	1.38–27.24	0.10–5.10	0.15–3.45
Magnesia (MgO)	0.20–3.70	0.25–3.00	0.25–2.86
Loss on ignition (LOI)	1.76–15.21	1.24–4.70	1.40–5.61
Sodium oxide (Na_2_O)	0.14–1.50	0.17–1.72	0.21–5.20
Sodium trioxide (SO_3_)	0.10–2.53	0.20–2.66	0.34–0.67

**Table 3:** Statistical measures of input features and target variable.

Statistic	Agro-Based Pozzolan	Cementitious Additive	PI	LL	PL	Duration	UCS
	(%)	(%)	(%)	(%)	(%)	(Days)	kPa
Mean	8.22	5.03	27.92	53.64	25.72	16.30	893.12
Standard Deviation	5.93	2.54	11.72	15.45	7.78	9.40	623.42
Kurtosis	−0.73	−0.61	−1.02	−1.05	−1.50	−1.71	−0.16
Skewness	0.64	0.42	0.78	0.62	0.12	0.31	1.63 × 10^−^^6^
Minimum	0.10	0.50	14.00	35.00	14.00	7.00	60.00
Maximum	25.00	11.25	50.00	86.00	37.00	28.00	3306.51

**Table 4:** Range of values of strength category for UCS.

Strength Category	UCS (kPa)
Very soft	<25
Soft	25–50
Firm	50–200
Stiff	200–400
Hard	>400

**Table 5:** Optimised parameters of GB algorithm.

Category	Parameter
Tree-based	min_samples_split = 6; max_depth = 5; min_samples_leaf = 11; max_leaf_nodes = 100; max_features = sqrt; min_weight_fraction_leaf = 0.005
Boosting	Subsmaple = 0.7999905; n_estimators = 501; Learning rate = 0.0805;
General functionality	loss = huber; random state = 20; criterion = friedman_mse; min_impurity_decrease = 8.999999999; init = None; alpha = 0.9; n_iter_no_change = None

**Table 6:** Multinomial classification metrics.

Multiclass Metric	Type	Value
Accuracy	Overall	0.920
	Weighted	0.966
AUC	Macro	0.951
	Micro	0.980
	Weighted	0.958
f1 score	Macro	0.818
	Micro	0.920
	Weighted	0.915
Precision	Macro	0.882
	Micro	0.920
	Weighted	0.938
Recall	Macro	0.833
	Micro	0.920
	Weighted	0.920

**Table 7:** ML performance of tree ensembles on the stabilised soil dataset.

Model	Optimised Parameters	R^2^		MAPE	
		Training	Testing	Training	Testing
Decision Tree	splitter = ‘random’, random_state = 51, max_depth = 20, min_samples_split = 5, criterion = ‘squared_error’, min_samples_leaf = 2, max_features = ‘auto’, min_impurity_decrease = 0.9, max_leaf_nodes = 50	0.820	0.813	0.332	0.915
Random Forest	n_estimators = 500, random_state = 5, max_depth = 9, min_samples_split = 5, criterion = ‘squared_error’, min_samples_leaf = 2, max_features = ‘log2’, oob_score = False, bootstrap = True, max_leaf_nodes = 100	0.861	0.827	0.328	0.845
Extremely Randomised Trees	n_estimators = 100, random_state = 90, max_depth = 9, min_samples_split = 6, criterion = ‘squared_error’, min_samples_leaf = 2, max_features = ‘auto’, oob_score = False, bootstrap = False, max_leaf_nodes = 70	0.867	0.849	0.211	0.513
Adaboost	base_estimator = Decision tree (max_depth = 7), n_estimators = 20, random_state = 45, loss = ‘exponential’, learning_rate = 0.9205	0.867	0.856	0.200	0.478
Gradient Boosting (this study)	Table 5	0.900	0.900	0.213	0.335

## Data Availability

Not applicable.
